# Therapeutic misconceptions: the protective role of the perioperative team

**DOI:** 10.1016/j.bjane.2025.844720

**Published:** 2025-12-25

**Authors:** Britta S. von Ungern-Sternberg, Aine Sommerfield

**Affiliations:** a Perth Children’s Hospital, Department of Anaesthesia and Pain Medicine, Child and Adolescent Health Service, Nedlands, Western Australia, Australia; b The University of Western Australia, Institute for Paediatric Perioperative Excellence, Anaesthesia and Pain Management Program, Perth, Western Australia, Australia; c The University of Western Australia, Division of Emergency Medicine, Anaesthesia and Pain Medicine, Perth, Western Australia, Australia; d The Kids Research Institute Australia, Perioperative Medicine Team, Nedlands, Western Australia, Australia

Informed research consent, by participants or their legal guardian, is a vital cornerstone of medical ethics, upholding patient autonomy and protecting research participants.^1^ However, ethical conduct by researchers in obtaining signed consent, including interactions between researchers and their patients, and in case of minors or other vulnerable groups, also their families and/or guardians, is vital to ensure that the research participant is not only educated about the research, but also fully comprehends the information as it pertains to them including interventions, possible risks and benefits as well as handling of their personal data and if applicable, collection and storage of samples. Only then can the participants make a voluntary, truly informed decision to participate without coercion or undue influence from outside.

Patients, particularly minors, undergoing surgical procedures represent a vulnerable patient population who may experience a significant power imbalance with their treating clinical team, who are often also the research team, which could influence their decision to participate. The perioperative period has been highlighted by patients and their families as a stressful time with high levels of anxiety,^2^^,^^3^ which can lead to a stress-induced decline in a patient’s rational comprehension.^4^ Emotional and cognitive distress, coupled with the hope for a cure or relief from their underlying illness, for which they are undergoing surgery, can increase the risk of a patient and/or, their families and/or guardians attributing a stronger personal benefit to the research than is really the case. The emotional desire for a positive outcome, a cure or simply symptom improvement can hinder full understanding of the research and make it even more difficult for patients to appreciate where standard clinical care ends and research begins. Patients and/or their families may also not comprehend that, in a randomized controlled trial, they may be assigned to a placebo and assume they will receive the active intervention if they participate.

Patients and/or their families may have an inappropriate belief, that their participation in research is primarily to provide an individual therapeutic benefit to them rather than to generate new knowledge for the benefit of future patients; this is called therapeutic misconception.^5^^,^^6^ The incidence of therapeutic misconception is assumed to be 50%‒75%^7^ and it undermines the validity of any informed consent.

## Causes of therapeutic misconceptions

Generally, the cognitive frames between researchers and patients/families differ fundamentally. The researcher’s mindset is scientific and objective, focused on answering a research question and generating new knowledge. In contrast, the patient’s cognitive frame is deeply personal, focusing on their individual health problems and outcomes, leading to a misinterpretation of study information. Simply adding more information to the informed consent documents is unlikely to mitigate this risk; it might exacerbate the overload.^8^

Therapeutic misconception is more likely when informed consent is performed by a person who holds dual roles, such as the treating physician and the study investigator.^1^^,^^9^ This is particularly important for patients with chronic diseases who are reliant on a long-term good patient-physician relationship and, therefore, might be particularly disinclined to decline a suggestion by their treating physician but instead feel obliged to consent to research. What steps can researchers take to mitigate the risk of therapeutic misconceptions by potential study participants and their families?

### Role of patient and public involvement

Patient and Public Involvement (PPI) also known as Consumer and Community Involvement (CCI) plays a vital role. It strengthens not only the acceptability of treatments, but it also improves trial design and the consenting process by taking patient/public preferences, values, as well as their concerns into consideration.^10^ Careful study design and wording of information and consent forms in close collaboration with patients and community members will improve the readability of patient documents, thereby enabling a better understanding of the research as well as reducing the risk of therapeutic misconceptions. For pediatric patients, it is important that there is not only a parent/guardian information sheet but also an age-appropriate child and/or young adult information sheet, which has been developed in collaboration with consumers of all ages to ensure readability. When age-appropriate, assent should be sought from all children for the intervention in addition to parental consent.

## Role of all perioperative team members

Anesthesiologists and other members of the perioperative team can play a critical role in minimizing the risks of therapeutic misconceptions and avoiding blurring of the lines between routine clinical care and research. As a vital foundation of good clinical research practice, there should be a focus on two-way communication around the research, rather than an over-reliance on the written informed consent process and the signing of the forms. Good communication may include plain language, visual aids or interactive media. The researcher should ensure, through conversation and questioning, that patients and if applicable their families/guardians understand what they are consenting to. Incorporating a teach back approach during the research consent process may help to reduce the risk of therapeutic misconception.

Dual roles as researchers and clinicians for the same patient should be avoided, if possible, to prevent subtle, even subconscious, coercion of patients into participation through the (direct or indirect) suggestion that participation is part of their personalized medical care. Not appreciating or failing to correct a perception that participation in the research is (in most cases) not part of a patient’s personalized medical care is also problematic. An independent, well-trained consent provider may significantly reduce these risks. Dual roles can be even more problematic if there is no clear delineation between research and routine clinical care. Is the suggested treatment or intervention “cutting-edge” clinical care or research? In some cases, such as oncology, it may be difficult even for members of the Ethics Committee or clinical colleagues to determine where routine clinical care ends and research begins, let alone for the patient and their family. Clear delineation is crucial by highlighting both pathways, including deviations from any national and/or international standards, in ethics applications and to all patients and their families.

This is where the close working relationship between all perioperative disciplines is advantageous: perioperative clinicians usually have a very close working relationship with their multi-disciplinary colleagues. We share the care of our patients, we regularly discuss perioperative management plans, we learn from each other in theatre and often discuss treatment options or cases. We are one team caring for the patient. While we certainly do not have the same specialist understanding of other perioperative specialties, physicians of all perioperative craft groups often have a deep insight into what is standard of care for certain patient groups. It is important that we use this knowledge to question when we are unsure whether the lines between clinical routine care and research may have been blurred in our craft group or others.

At the time of sign-in to theatre, all surgical/interventional consents are checked by the anesthesiologist before inducing anesthesia. This check should be performed in an environment where the patient and/or their family/guardian feels comfortable asking questions without time pressure, fear of reprisal or jeopardizing their care. It is good clinical practice to not only check the signature is in the right place and the form in date but to also check the correct understanding of the patient and/or their carer/guardian about the procedure and to ensure all relevant questions have been answered. The same principle should apply to any consent to participate in perioperative research. Does the patient truly understand what they have consented to and what the research entails? Do they understand any differences from routine clinical care? In our institution, we have a huddle before the start of each operating list, where all patients and their management are discussed, including any participation in research within the current perioperative visit. To aid this, the research teams must provide a copy of the signed consent sheet plus the participant information form and an institutional document which is a brief description (one paragraph) of the study interventions/observations highlighting the duration of participation and potential implications on clinical care e.g., side effects, drug interactions. Such an approach provides opportunities for perioperative colleagues to be made aware of the research, to remind everyone of any study requirements and any potential therapeutic misconceptions. Peer-to-peer collaboration and feedback can help to improve perioperative communication and ensure that all research processes, including consent, are transparent and that all information is understood.

Furthermore, this is not only good clinical practice ensuring patient safety, and high-quality data collection in an ethical environment, but it is, in our experience, also a good starting point for further conversations (after the case in the break room), to discuss the research and to brainstorm further ideas to be explored. It spreads the word beyond just academic colleagues and supports an academic culture in everyday clinical practice.

Such an open and transparent system must include protections for any clinicians who speak up about potential therapeutic misconceptions. Speaking up for safety is not always easy and/or safe.^11^^,^^12^ Chief investigators are often senior figures within the hospital who commonly have significant institutional support. Hierarchical structures may further complicate speaking up, particularly when institutions are fearful of a powerful perpetrator or wary of potential negative media. This may lead to institutional silence or inaction, leaving the problem unresolved. This can lead to the normalization of questionable or unethical behavior, leading to a decline in overall institutional culture and possibly to moral distress or moral injury of staff involved.^13^ So how should we speak up? This very much depends on the situation, the players involved and the urgency to speak up (e.g., is there a risk for immediate patient harm?). In our experience, there is no one-size-fits-all approach. If oneself is not sure about the intervention, a good starting point would be to speak directly in a non-judgmental manner with a member of the research team in a quiet environment. A friendly request seeking further information about the planned intervention and/or the rationale can start the conversation. This allows the colleague to give further background information and explain their reasoning, which most of the time will resolve the problem. Similarly, if there is any suspicion that the patient and their family may be at risk for a therapeutic misconception, simply highlighting the fact of a potential misunderstanding on the part of the patient/family to the colleagues involved, and asking them to clarify with the patient and/or their family is most often received with gratitude. The great majority of clinicians try their very best to give their patients a true picture of the planned research interventions. However, if this non-confrontational approach is not received in a positive way and/or there may be the potential for direct patient harm, reporting lines should be used to escalate the situation.

In conclusion, therapeutic misconceptions are unfortunately not uncommon in clinical research; however, in the perioperative environment, we can leverage a higher degree of peer feedback and control to optimize our communication and consent processes for clinical research. We must also ensure that any staff who speak up about potential therapeutic misconceptions are kept safe and protected.

## Declaration of competing interest

The authors declare no conflicts of interest.
